# Combination of proviral and antiviral roles of B cell-intrinsic STAT1 expression defines parameters of chronic gammaherpesvirus infection

**DOI:** 10.1128/mbio.01598-24

**Published:** 2024-10-23

**Authors:** Erika R. Johansen, Damon L. Schmalzriedt, Danilela Avila, Paul A. Sylvester, Cade R. Rahlf, Jordan M. Bobek, Daisy Sahoo, Bonnie N. Dittel, Vera L. Tarakanova

**Affiliations:** 1 Department of Microbiology and Immunology, Medical College of Wisconsin, Milwaukee, Wisconsin, USA; 2 Department of Biochemistry, Medical College of Wisconsin, Milwaukee, Wisconsin, USA; 3 Department of Medicine, Medical College of Wisconsin, Milwaukee, Wisconsin, USA; 4 Cardiovascular Center, Medical College of Wisconsin, Milwaukee, Wisconsin, USA; 5 Cancer Center, Medical College of Wisconsin, Milwaukee, Wisconsin, USA; 6 Versiti Blood Research Institute, Milwaukee, Wisconsin, USA; The University of North Carolina at Chapel Hill, Chapel Hill, North Carolina, USA

**Keywords:** STAT1, interferon, gammaherpesvirus, chronic infection, germinal center, B cell differentiation, latency

## Abstract

Gammaherpesviruses are species-specific, ubiquitous pathogens that establish lifelong infection in their hosts and are associated with cancers, including B cell lymphomas. Type I and II interferons (IFNs) are critical for the control of acute and chronic gammaherpesvirus infection. However, the cell type-specific role of IFN signaling during natural infection is poorly defined and is masked by the altered viral pathogenesis observed in hosts with global IFN deficiencies. STAT1 is a constitutively expressed transcription factor that is critical for the effector function of type I and II IFNs. In this study, we defined the impact of B cell-specific STAT1 expression on gammaherpesvirus infection using murine gammaherpesvirus 68 (MHV68). While the acute stage of MHV68 infection was not affected, we found opposite, anatomic site-dependent effects of B cell-intrinsic STAT1 expression during chronic infection. Consistent with the antiviral role of STAT1, B cell-specific STAT1 expression attenuated the latent viral reservoir in peritoneal B cells of chronically infected mice. In contrast, STAT1 expression in splenic B cells supported the establishment of the latent MHV68 reservoir in germinal center B cells, revealing an unexpected proviral role of B cell-intrinsic STAT1 expression during chronic gammaherpesvirus infection. These STAT1-dependent MHV68 chronic infection phenotypes were fully recapitulated in the peritoneal cavity but not the spleen of mice with B cell-specific deficiency of type I IFN receptor. In summary, our study uncovers the intriguing combination of proviral and antiviral roles of B cell-intrinsic STAT1 expression during chronic gammaherpesvirus infection.

**IMPORTANCE:**

Interferons (IFNs) execute broadly antiviral roles during acute and chronic viral infections. The constitutively expressed transcription factor STAT1 is a critical downstream effector of IFN signaling. Our studies demonstrate that B cell-intrinsic STAT1 expression has opposing and anatomic site-dependent roles during chronic gammaherpesvirus infection. Specifically, B cell-intrinsic STAT1 expression restricted gammaherpesvirus latent reservoir in the peritoneal cavity, consistent with the classical antiviral role of STAT1. In contrast, decreased STAT1 expression in splenic B cells led to the attenuated establishment of gammaherpesvirus latency and decreased latent infection of germinal center B cells, highlighting a novel proviral role of B cell-intrinsic STAT1 expression during chronic infection with a B cell-tropic gammaherpesvirus. Interestingly, B cell-specific type I IFN receptor deficiency primarily recapitulated the antiviral role of B cell-intrinsic STAT1 expression, suggesting the compensatory function of B cell-intrinsic type II IFN signaling or an IFN-independent proviral role of B cell-intrinsic STAT1 expression during chronic gammaherpesvirus infection.

## INTRODUCTION

Gammaherpesviruses, including the two human gammaherpesviruses, Epstein–Barr virus (EBV) and Kaposi’s sarcoma-associated herpesvirus (KSHV), are ubiquitous pathogens that are associated with several cancers, including B cell lymphomas (1). Inadequate control of chronic infection, as defined by an increased viral latent reservoir and reactivation, is a prerequisite for gammaherpesvirus-driven tumorigenesis (2–8). However, there is insufficient mechanistic understanding of the host factors that attenuate chronic EBV and KSHV infections or are usurped by gammaherpesviruses to support the latent reservoir and viral reactivation in the intact host. To overcome the challenges of studying chronic EBV and KSHV infections, this study uses murine gammaherpesvirus 68 (MHV68), a rodent pathogen that offers a powerful animal model of chronic gammaherpesvirus infection of a natural intact host and shares key features with its human counterparts, including genetic conservation (9–11), B cell lymphomagenesis (8, 12), and manipulation of B cell differentiation during chronic infection (13, 14).

A unique feature of gammaherpesviruses is their ability to usurp host B cell differentiation to support the establishment of the chronic latent reservoir in memory B cells (14–19). Specifically, EBV and MHV68 infect naïve B cells and drive the germinal center response where both infected and bystander B cells rapidly proliferate and differentiate into memory B cells that house lifelong latent reservoirs, or plasma cells that support viral reactivation (20, 21). In addition to supporting the expansion of the latent viral reservoir, infected germinal center B cells are thought to be the target of lymphomagenesis as most EBV-positive lymphomas are germinal center- or post-germinal center-derived (8). Despite the importance of germinal center response in natural infection and pathogenesis, the host and viral factors driving the germinal center response during EBV infection are poorly understood largely due to the exquisite species specificity of human gammaherpesviruses and the inability to model germinal center response *in vitro*. To overcome this limitation, the MHV68 experimental model has served as a tractable tool to identify several host and viral factors that support or attenuate gammaherpesvirus-driven germinal center responses (15, 22–33).

Germinal center-driven B cell differentiation is largely limited to B-2 B cells that undergo a well-defined development program in the bone marrow with subsequent differentiation in secondary lymphoid organs. In addition to the infection and manipulation of B-2 B cells in the secondary lymphoid organs, MHV68 also infects B-1 B cells in the peritoneal cavity. B-1 B cells represent a unique primordial B cell lineage that develops in the embryonic yolk sac, self-renews, and primarily resides in body cavities, with transient and limited circulation through the secondary lymphoid organs (34). The B-1 B cell lineage is further subdivided into B1a- and B1b-B cell populations. While B1a-B cells are not latently infected by MHV68, the majority of peritoneal latent reservoir at 16 days post-intranasal inoculation is housed by B1b B cells, with some latent infection also observed in B-2 B cells residing in the peritoneal cavity (35). The significance of B-1 B cell infection for the maintenance of lifelong gammaherpesvirus infection and viral pathogenesis remains unclear. However, it is intriguing that KSHV-driven primary effusion B cell lymphoma (PEL) is almost exclusively located in the body cavities and not the secondary lymphoid organs where other types of B cell lymphomas are found (36). However, the genesis of PEL remains unclear due to the unique combination of phenotypic and genetic B cell markers displayed by malignant cells.

Type I, II, and III interferons (IFNs) represent an important antiviral defense of the host. IFNs engage cognate receptors to stimulate signaling cascades that induce the activation of STAT1, a constitutively expressed transcription factor that executes classical effector functions of IFNs. STAT1-containing transcription complexes induce the expression of hundreds of interferon-stimulated genes (ISGs), with unique combinations of ISGs demonstrating antiviral or proviral functions for specific viral families (37, 38). Despite multiple gammaherpesvirus proteins reported to antagonize IFN signaling in cultured cells, systemic type I and II IFN signaling plays a critical role in the attenuation of acute and chronic gammaherpesvirus infection in an intact host. Specifically, *Ifnar1^−/−^* mice that lack the expression of type I IFN receptor have profoundly altered pathogenesis of MHV68 infection with significant acute mortality and high levels of persistent lytic replication (for at least 3 weeks) in the survivors of acute infection (39–41). Furthermore, the administration of IFNAR1-blocking antibodies to chronically infected wild-type mice is sufficient to trigger MHV68 reactivation (41).

Effects of global type II IFN deficiency on acute MHV68 infection are virus dose-dependent and range from mild delay of lytic virus clearance in *Ifng^−/−^* mice infected with a lower [4 × 10^3^ plaque-forming units (PFU)] dose of MHV68 to acute lethal pneumonia in *Ifng^−/−^* mice infected with a 100-fold higher dose of MHV68 (42, 43). Importantly, global deficiency of type II IFN signaling profoundly changes the pathogenesis of chronic infection. Increased MHV68 reactivation and persistent replication in type II IFN-deficient mice, concurrent with a largely futile antiviral T cell response, lead to multiple organ fibrosis and large vessel arteritis with chronic morbidity and eventual mortality (44–47). The effects of type III IFN are mostly limited to epithelial cells due to the limited receptor expression and are poorly understood in MHV68 infection. In a single study (48), overexpression of IFNλ did not affect MHV68 reactivation from the genital mucosa; the effects of type III IFN deficiency on MHV68 infection are unknown.

While valuable insights into the control of gammaherpesvirus infection have been obtained using mice with global type I or type II IFN deficiencies, the lack of global IFN signaling leads to the profound alteration of the balance between latent and lytic MHV68 life cycles and viral pathogenesis. Furthermore, given the concurrent expression of type I, II, and likely III IFNs during chronic MHV68 infection, it is impossible to define the combined effect of global IFN responses without eliminating the infected host acutely, as evidenced by the uniform and acute mortality of *Stat1^−/−^* mice infected with a low (100 PFU) dose of MHV68 (41). Finally, global IFN deficiency makes it challenging if not impossible to define cell type-specific effects of IFN signaling during gammaherpesvirus infection of an intact host.

To address the challenges described above, our published and current studies have employed genetic approaches to target STAT1, a critical effector of all classical IFN responses, in cell types relevant to MHV68 infection. Using a combination of viral and host genetic approaches, we uncovered the myeloid-selective antiviral role of STAT1 that is antagonized by the conserved MHV68 protein kinase during the establishment of chronic infection (49). In contrast, we discovered that T cell-intrinsic STAT1 expression has an overall proviral effect during chronic MHV68 infection (50), an effect that must be indirect as MHV68 does not infect T cells. Capitalizing on the advantages offered by the MHV68 system, the current study uses the genetic host model where conditional STAT1 alleles (51) are targeted by CD19 promoter-driven Cre recombinase to define the role of B cell-specific STAT1 expression during gammaherpesvirus infection of an intact host.

Consistent with the classical antiviral role of STAT1, B cell-intrinsic STAT1 deficiency led to increased latent MHV68 reservoir in peritoneal B cells. In contrast, we observed that B cell-specific STAT1 deficiency resulted in reduced latent infection of germinal center B cells and attenuated MHV68-driven germinal center response, unveiling a novel proviral role of B cell-intrinsic STAT1 expression during chronic gammaherpesvirus infection. To define the extent to which B cell-intrinsic type I IFN signaling was responsible for the observed viral and host phenotypes, we generated a mouse model of B cell-specific type I IFN receptor deficiency. Interestingly, attenuated type I IFN signaling in B cells fully recapitulated MHV68 phenotypes observed in the peritoneal cavity, but not the spleen of mice with B cell-specific STAT1 deficiency, suggesting that type I IFN signaling in peritoneal B cells plays a non-redundant antiviral role during chronic gammaherpesvirus infection.

## RESULTS

### Generation of B cell-specific STAT1 deficiency mouse model

**Fig 1:**
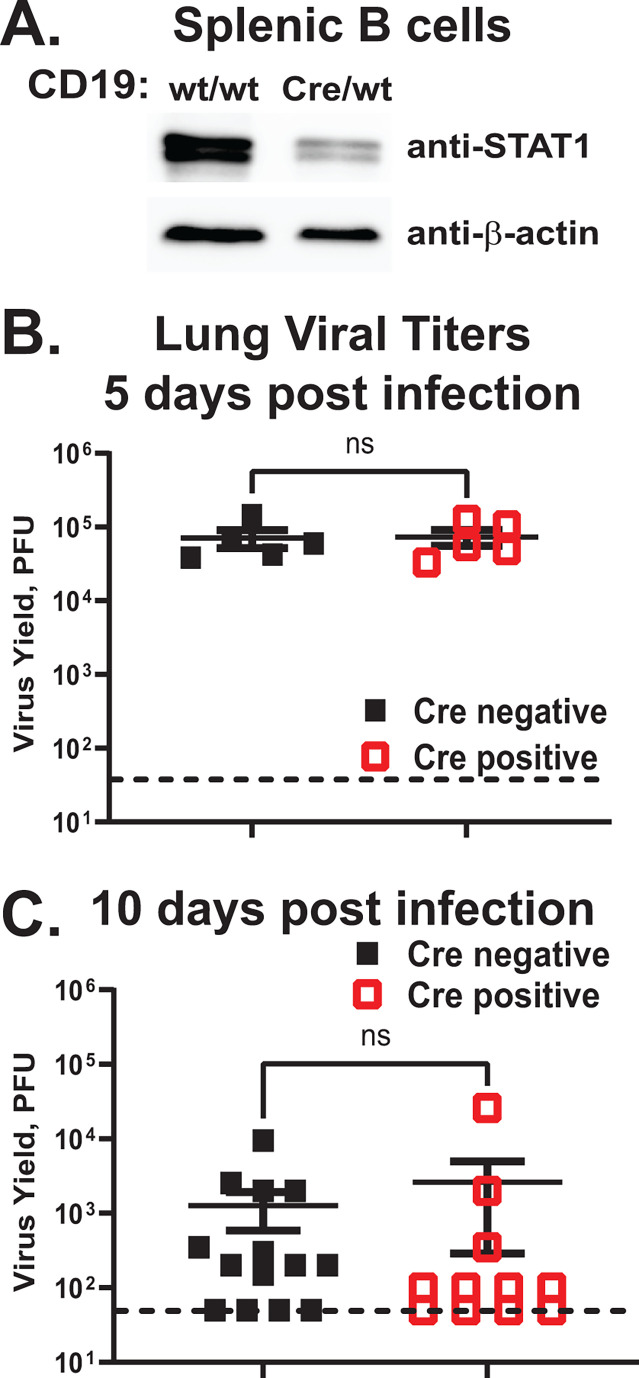
Acute MHV68 replication is not affected by B cell-specific STAT1 deficiency. (A) Lysates from splenic B cells sorted from CD19 Cre-negative and -positive STAT1^fl/fl^ littermates were subjected to western analyses using indicated antibodies. (B, C) CD19 Cre-negative and -positive STAT1^fl/fl^ littermates were intranasally infected with 10,000 PFU of MHV68. Lungs harvested at 5 (**B**) or 10 (**C**) days post-infection were homogenized and subjected to plaque assay to measure MHV68 titers. Each symbol indicates an individual mouse. Dotted line indicates limit of detection. Here and in subsequent figures, error bars represent the standard error of the mean (SEM).

Given the profoundly altered pathogenesis of MHV68 infection in mouse models of global type I or type II IFN deficiency and the critical effector role of STAT1 downstream of all IFN receptors, we sought to generate a mouse model with all classical IFN signaling specifically attenuated in B cells. To achieve a B cell-specific STAT1 deficiency model, CD19-Cre recombinase knock-in allele (52) was crossed to the conditional *Stat1* allele (51) to generate *Cd19^Cre/wt^ Stat1^fl/fl^* and *Cd19^wt/wt^ Stat1^fl/fl^* littermates (referred to as Cre-positive and Cre-negative, respectively). Analyses of sorted splenic B cells showed a decrease in STAT1 protein levels in B cells of Cre-positive mice (Fig. 1A). Baseline levels of peripheral B cell populations (transitional, marginal zone, and follicular) and peritoneal B cells were not altered by the B cell-intrinsic STAT1 deficiency (Fig. S1 and S2), indicating that B cell-specific STAT1 deficiency does not affect B cell development.

### Acute MHV68 replication is not affected by B cell-specific STAT1 deficiency

Following the inoculation of a naïve host, MHV68 undergoes lytic replication during the first 10–12 days post-infection. While MHV68 primarily infects lung epithelial cells and macrophages during the peak phase of acute infection (53), by 16 days post-infection, a majority of MHV68 DNA-positive cells in the lung are represented by CD19^+^ B cells (54). The effect of B cell-intrinsic STAT1 deficiency on acute MHV68 replication was first measured at 5 days post-infection, at the peak of MHV68 replication. Consistent with MHV68 tropism for epithelial cells and macrophages at this time post-infection, similar MHV68 titers were observed in the lungs of Cre-negative and Cre-positive littermates (Fig. 1B). Given the transition of MHV68 infection to lung B cells by 16 days post-infection, MHV68 acute titers were measured again, at 10 days post-infection, when acute viral replication is being cleared. Similar to that observed during peak MHV68 replication, lung viral titers were similar in Cre-negative and Cre-positive groups at 10 days post-infection (Fig. 1C, *P* = 0.22). Thus, B cell-intrinsic STAT1 deficiency did not affect acute MHV68 replication in the lungs under the experimental conditions used.

### B cell-intrinsic STAT1 expression has anatomic site-specific roles in the regulation of chronic gammaherpesvirus infection

**Fig 2:**
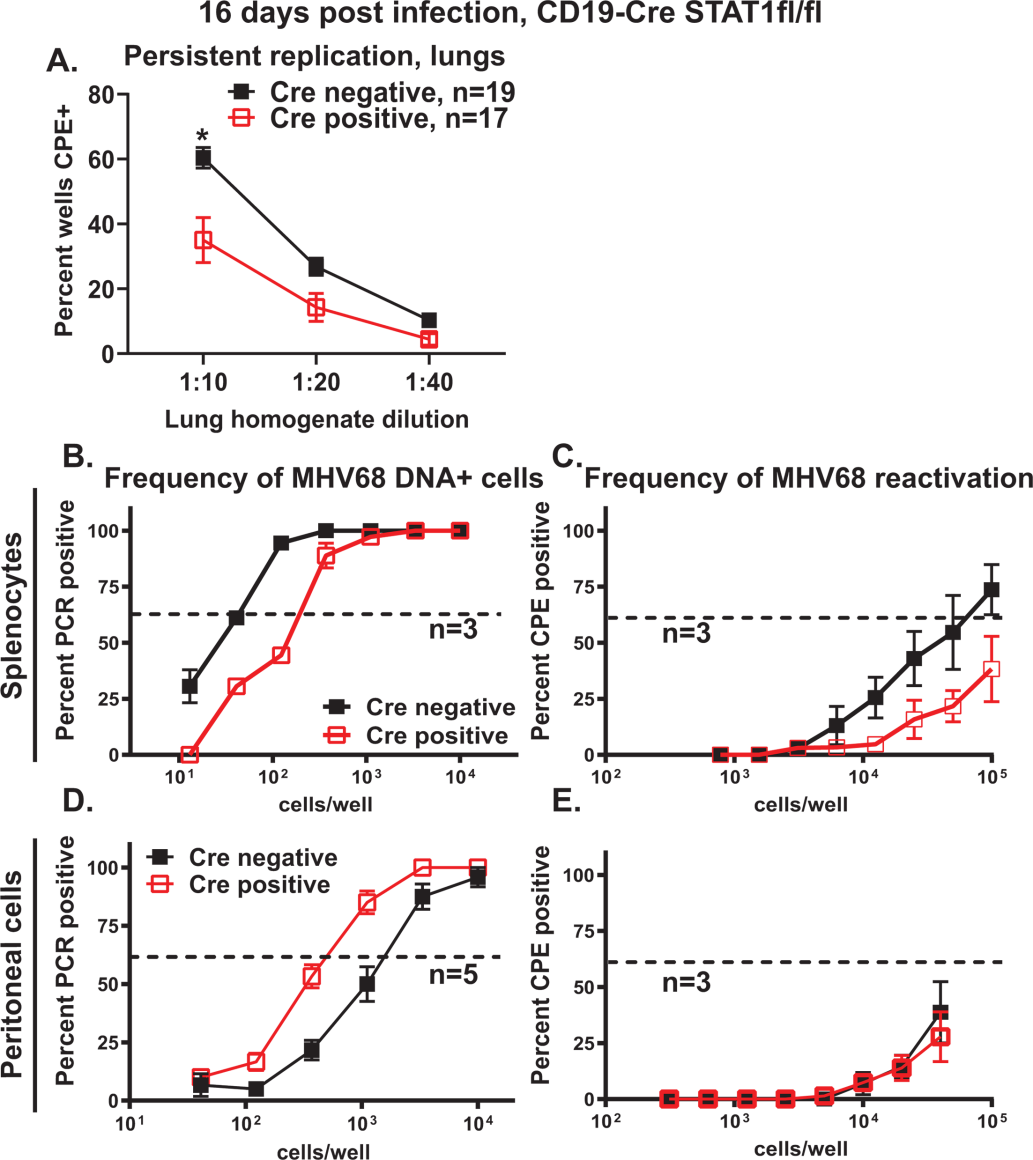
B cell-intrinsic STAT1 expression has anatomic site-specific roles in the regulation of chronic gammaherpesvirus infection. CD19 Cre-negative and -positive STAT1^fl/fl^ littermates were infected as in Fig. 1 and analyzed at 16 days post-infection. (A) Persistent MHV68 replication in lung homogenates. Data were pooled from indicated number of animals in each group. ^*^P < 0.05. Splenocytes (**B, C**) and peritoneal cells (**D, E**) were pooled from three to five animals/group in each study and cell suspensions subjected to limiting dilution assays to define the frequency of MHV68 DNA+ cells (**B, D**) and frequency of *ex vivo* reactivation (**C, E**). Data were pooled from indicated number (*n*) of independent studies. In the limiting dilution assays, the dotted line is drawn at 63.2%, and the *x*-coordinate of the intersection of this line with the sigmoid graph represents an inverse of frequency of positive events.

Along with clearance of acute lytic MHV68 replication by 12 days post-infection, there is a concomitant rise in the latent viral reservoir that peaks at 16 days post-infection in the spleen (55). However, despite the systemic clearance of most infectious virions, very low levels of persistent MHV68 replication are maintained in the lungs for at least the first month post-infection (56). To define the role of B cell-intrinsic STAT1 expression during persistent MHV68 replication, levels of preformed infectious MHV68 were measured in the lungs of Cre-negative and Cre-positive littermates at 16 days post-infection. Persistent replication was measured using a highly sensitive, mouse embryonic fibroblast (MEF)-based semi-quantitative assay designed to detect very low levels of lytic MHV68 that would not be detected by the conventional plaque assays. Interestingly, infectious MHV68 levels were reduced in Cre-positive lungs (Fig. 2A, data pooled from the indicated number of animals per genotype), indicating that B cell-intrinsic STAT1 expression supports persistent MHV68 replication in the lungs.

The spleen and peritoneal cavity are the two best-studied anatomic sites that support the MHV68 latent reservoir, with the peak of the splenic latent reservoir observed at 16 days post-infection. Interestingly, B cell-intrinsic STAT1 deficiency led to contrasting effects on the MHV68 chronic infection at the two anatomic sites. Specifically, the frequency of MHV68 DNA-positive splenocytes was decreased 4.6-fold in Cre-positive mice, along with a similar decrease in the frequency of *ex vivo* MHV68 reactivation (Fig. 2B and C). In contrast, the frequency of MHV68 DNA-positive peritoneal cells was increased 3.2-fold in Cre-positive mice, with similarly low levels of *ex vivo* reactivation observed in peritoneal cells of both groups (Fig. 2D and E). Persistent MHV68 replication was not detected in splenocytes and peritoneal cells, regardless of the genotype (data not shown). In summary, B cell-intrinsic STAT1 expression demonstrated an unexpected proviral role in chronic MHV68 infection, as it supported persistent MHV68 replication in the lungs and the establishment of MHV68 latency in the spleen. In contrast, the traditional antiviral function of STAT1 was observed in the peritoneal cavity, where B cell-intrinsic STAT1 expression attenuated the establishment of MHV68 latency in peritoneal cells.

### B cell-intrinsic STAT1 expression supports the MHV68-driven germinal center response and selectively promotes MHV68-driven increase in self-reactive antibodies

**Fig 3:**
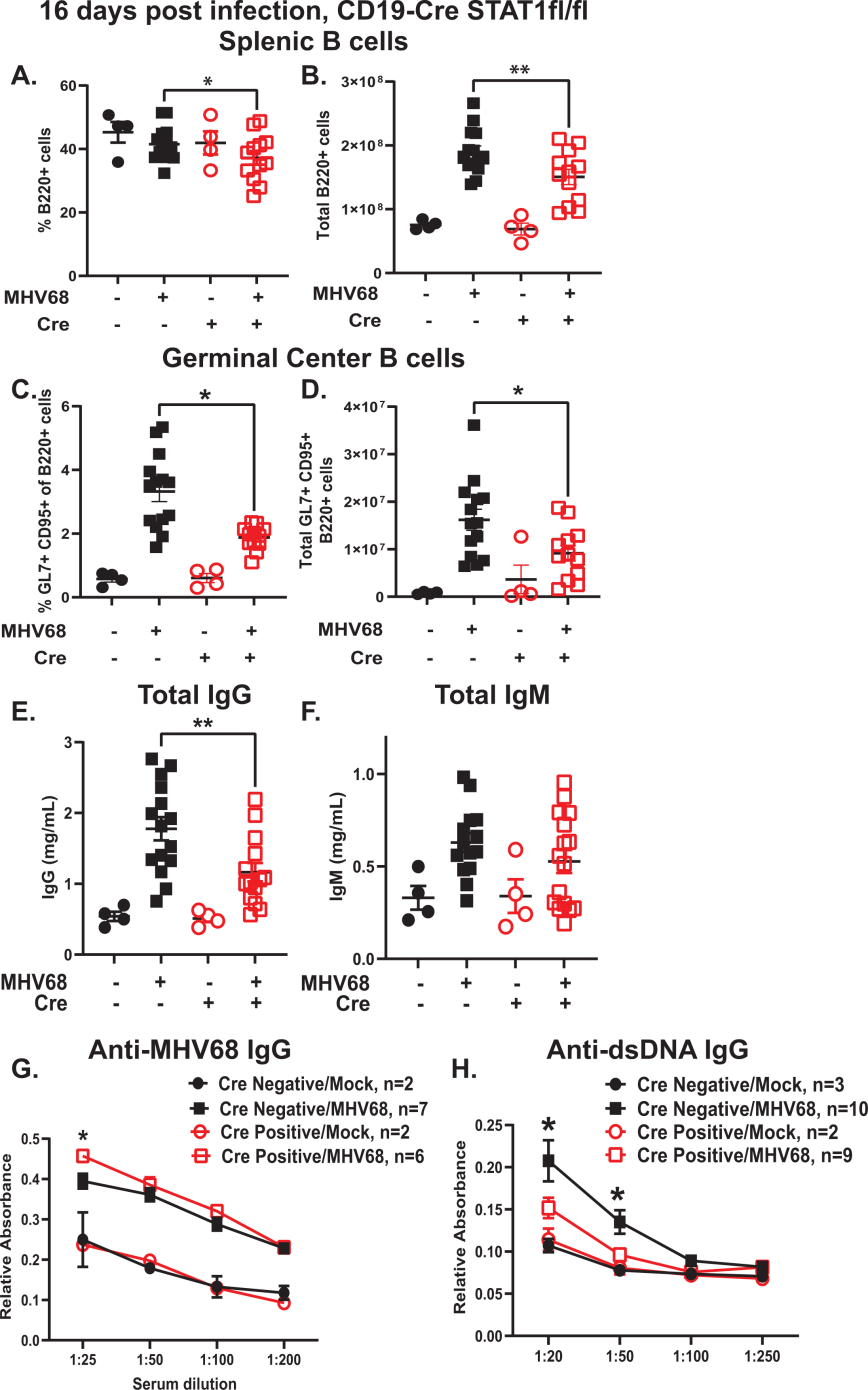
B cell-intrinsic STAT1 expression supports MHV68-driven germinal center response and selectively promotes generation of MHV68-driven self-reactive antibodies. CD19 Cre-negative and -positive STAT1^fl/fl^ littermates were infected as in Fig. 1 and analyzed at 16 days post-infection. (A, B) Splenic B cells defined as B220^+^ (representative flow plots in Fig. S3) were measured by flow cytometry with proportion (**A**) and absolute cell number (**B**) shown. (C, D) Splenic germinal center B cells defined as B220^+^GL7^+^CD95^+^ were measured with proportion (**C**) and absolute cell number (**D**) shown. (E–H) Sera collected from infected and mock-infected mice of indicated genotypes at 16 days post-infection were subjected to ELISA to measure levels of total IgG (**E**), total IgM (**F**), anti-MHV68 IgG (**G**), and anti-double-stranded DNA IgG (**H**). Each symbol in (A–F) represents an individual animal. In (G and H), “*n*” indicates the number of sera from individual animals that were analyzed with data pooled within each group. ^*^P < 0.05; ^**^P < 0.01.

The establishment of a splenic MHV68 latent reservoir is intimately tied to B cell differentiation. Specifically, MHV68 latently infects naïve B cells, which are subsequently driven, along with uninfected counterparts, to enter the germinal center reaction, where the robust proliferation of germinal center B cells supports an increase in the gammaherpesvirus latent reservoir (14, 17, 19). Further differentiation of latently infected germinal center B cells into antibody-secreting plasmablasts and plasma cells triggers viral reactivation (20, 21). Having observed a decreased MHV68 latent reservoir and reactivation in Cre-positive splenocytes, the effect of B cell-specific STAT1 expression on the MHV68-driven germinal center response was examined next. Both the proportion and absolute numbers of overall splenic B cells were decreased in MHV68-infected Cre-positive mice at 16 days post-infection (Fig. 3A and B). Consistent with the decrease in the splenic latent reservoir, the proportion and absolute number of splenic germinal center B cells were also decreased in Cre-positive mice (Fig. 3C and D; gating strategy and representative flow plots in Fig. S3). CD4 T follicular helper cells (TFH) are critical for both physiological and MHV68-driven germinal center responses (15, 57). Consistent with decreased germinal center B cells, MHV68-infected Cre-positive mice also showed decreased proportion and absolute numbers of TFH (Fig. S4A through C). Thus, B cell-intrinsic STAT1 expression supported the MHV68-driven germinal center response during chronic infection.

Antibody class-switching preferentially occurs during germinal center-based B cell differentiation. Having observed a decreased MHV68-driven germinal center response in Cre-positive mice (Fig. 3C and D; Fig. S3A through C), antibody levels were measured next. As expected, serum titers of class-switched IgG antibodies were robustly induced by MHV68 infection in control Cre-negative mice (Fig. 3E). In contrast, the total IgG titers were significantly lower in sera of MHV68-infected Cre-positive mice. Unlike that observed for serum IgG titers, IgM titers were equally elevated by MHV68 infection in Cre-negative and Cre-positive mice (Fig. 3F).

In a physiological immune response, germinal center B cell differentiation during viral infection leads to the production of virus-specific antibodies with several effector functions. In contrast, the gammaherpesvirus-driven germinal center response differs from the physiological response, as the former supports both virus-specific and extensive self- and foreign species-directed B cell responses. As a result, there is a robust, albeit transient increase in the titers of antibodies reactive against diverse self- and foreign species antigens (58, 59). In fact, the presence of high titer antibodies against horse antigens is diagnostic of a recent EBV infection in adolescents and adults (60). The MHV68-driven antibody response against self-antigens, including double-stranded DNA, peaks at 14 days post-infection (59). In contrast, only a small proportion of antibody-secreting B cells at 14 days post-infection produce anti-MHV68 antibodies (61). Consequently, the rise in the titers of MHV68-specific class-switched antibodies is significantly delayed and does not peak until at least 3 weeks post-infection (58). Having observed a decrease in IgG serum titers in MHV68-infected Cre-positive mice (Fig. 3E), virus-specific and self-reactive class-switched antibodies were measured next. Despite the decrease in total IgG titers in Cre-positive mice, the levels of class-switched MHV68-specific IgG were not decreased (Fig. 3G) with a small statistically significant increase observed in the Cre-positive MHV68-infected group at the single, most concentrated serum dilution (Fig. 3G). As anti-MHV68 antibody does not play a role in the control of chronic infection of an immunocompetent mouse (62), the biological relevance of the observed increase is unclear.

While antibodies against diverse self-antigens are driven by MHV68 infection, the generation of class-switched antibodies against double-stranded DNA occurs in most MHV68-infected mice and represents a reliable marker of an otherwise highly heterogeneous self-reactive antibody response (59). In contrast to that observed for MHV68-specific antibody titers, the titers of anti-double-stranded DNA IgG were decreased in the Cre-positive as compared to Cre-negative mice following MHV68 infection (Fig. 3H). In summary, B cell-intrinsic STAT1 expression supported the MHV68-driven germinal center response and selectively supported the generation of self-reactive but not virus-specific antibody responses during MHV68 infection.

### B cell-intrinsic STAT1 expression supports MHV68 infection of germinal center B cells

**Fig 4:**
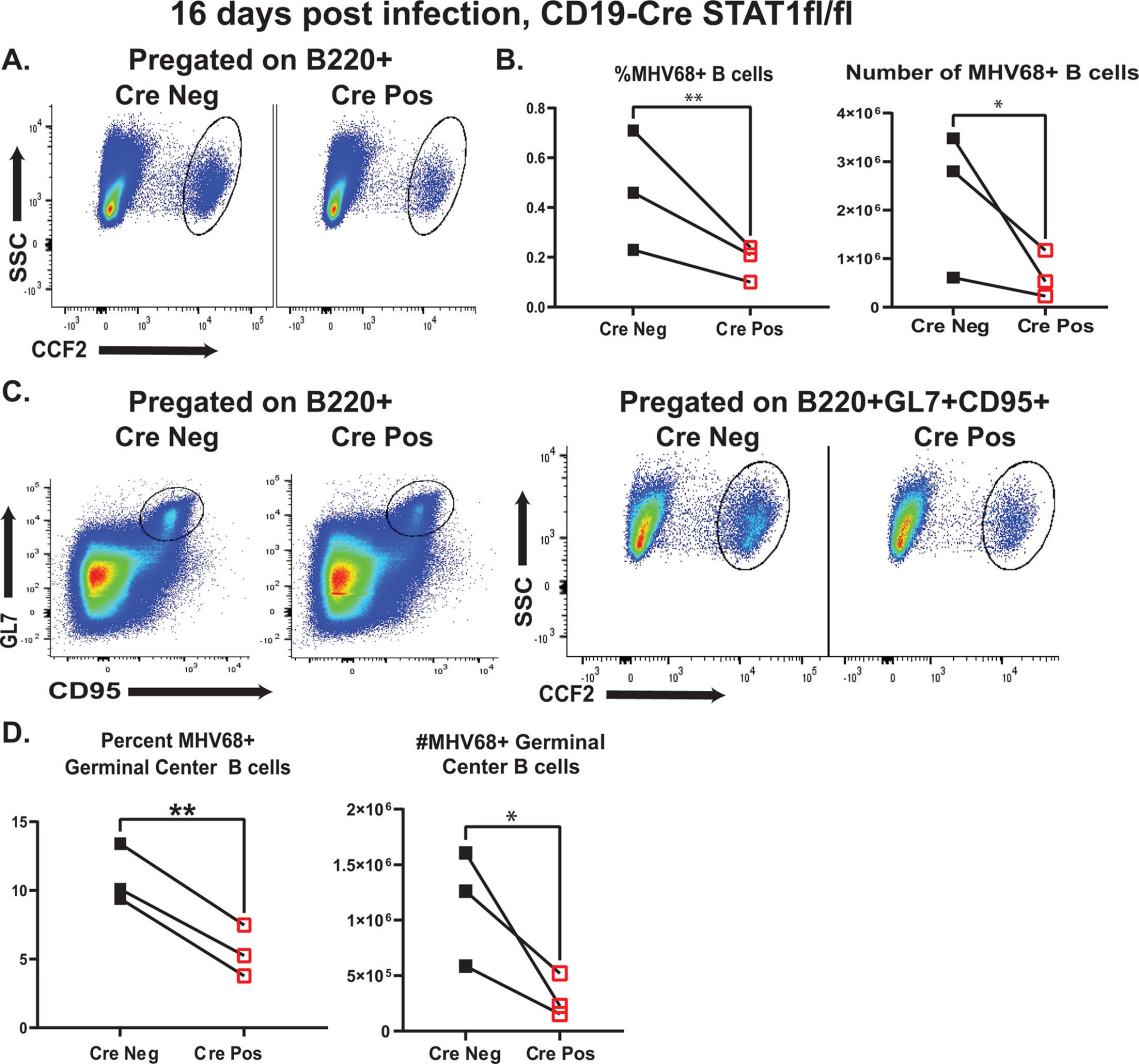
B cell-intrinsic STAT1 expression supports MHV68 infection of germinal center B cells. CD19 Cre-negative and -positive STAT1^fl/fl^ littermates were intranasally infected with 10,000 PFU of MHV68.ORF73βla reporter virus, spleens harvested at 16 days post-infection, and pooled from three animals within each group within each independent experiment. Infected cells were defined as positive for cleaved CCF2 β-lactamase substrate (indicated as CCF2^+^). (A) Representative gating strategy for CCF2^+^ B220^+^ splenocytes. (B) Percent and absolute number of infected splenic B220^+^ cells. (C) Representative gating strategy for germinal center B cells (B220^+^GL7^+^CD95^+^) subsequently gated for cleaved CCF2 substrate (CCF2^+^) to define infected germinal center B cells. (D) Percent and absolute number of infected germinal center B cells. In (B and D), each symbol represents analysis of splenocytes pooled from three to five mice/genotype in a single experiment; the connecting lines represent paired observations within a single study. Results of three studies are pooled. ^*^P < 0.05; ^**^P < 0.01.

Having observed an attenuated splenic latent reservoir and B cell differentiation in Cre-positive mice, the infection of germinal center B cells was examined next, as germinal center B cells house most of the splenic MHV68 latent reservoir at 16 days post-infection. To define the frequency and number of latently infected B cells, mice were infected with the MHV68.ORF73βla reporter virus that encodes a fusion of mLANA, an MHV68 latent protein critical for the viral episome maintenance, and β-lactamase (63), enabling the detection of latently infected cells by flow cytometry using a cell-permeable β-lactamase substrate (CCF2). The frequency and number of MHV68-positive splenic B cells were lower in Cre-positive as compared to Cre-negative mice (Fig. 4A and B), consistent with the decrease in the frequency of infected Cre-positive splenocytes determined by limiting dilution PCR assays (Fig. 2B). Importantly, the frequency and the absolute number of MHV68-positive germinal center B cells were also significantly lower in Cre-positive spleens (Fig. 4C and D), indicating that B cell-specific STAT1 expression supports effective latent MHV68 infection of germinal center B cells, in addition to supporting MHV68-driven germinal center response.

### B cell-intrinsic STAT1 expression attenuates latent infection of peritoneal B cells

**Fig 5:**
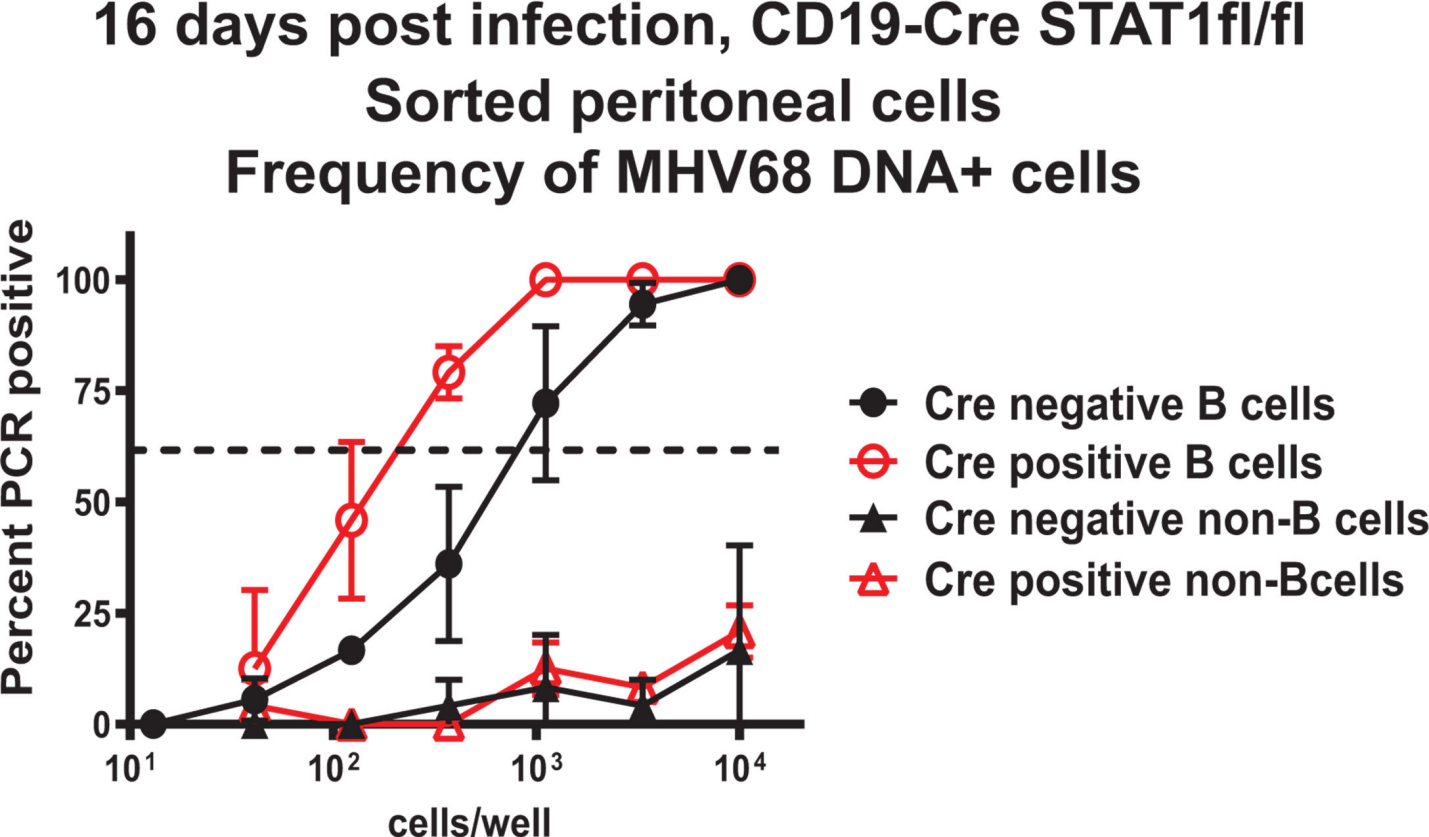
B cell-intrinsic STAT1 expression attenuates latent infection of peritoneal B cells. CD19 Cre-negative and -positive STAT1^fl/fl^ littermates were intranasally infected with 10,000 PFU of wild-type MHV68. At 16 days post-infection, peritoneal cells were pooled from three to five mice/group in each experiment, pooled cells sorted into CD19^+^ B cells and CD19^−^ non-B cells using magnetic beads, and sorted populations subjected to limiting dilution PCR assays. Data were pooled from two to three independent experiments. The dotted line is drawn at 63.2%, and the *x*-coordinate of intersection of this line with the sigmoid graph represents an inverse of frequency of positive events.

In contrast to the surprising proviral role of B cell-intrinsic STAT1 expression in the spleen, we observed an increase in the MHV68 latent reservoir in peritoneal cells harvested from chronically infected Cre-positive mice (Fig. 2D). We previously showed that following intranasal MHV68 inoculation (also used in this study), MHV68 latent infection is enriched in peritoneal B-1 B cells, with peritoneal B-2 B cells being a secondary cell type hosting latent reservoir (34). In contrast, at 16 days post-intranasal infection, CD11b^+^ myeloid cells and B220^−^CD19^−^CD11b^−^ peritoneal cells do not play a significant role in hosting MHV68 latent reservoir in the peritoneal cavity. This viral tropism is modified by the inoculation route, as intraperitoneal MHV68 inoculation leads to the peritoneal myeloid cells hosting most of the latent MHV68 (34). Body cavity resident B-1 B cells are developmentally and functionally distinct from the B-2 B cells, with the latter residing in and circulating through the secondary lymphoid organs, including the spleen. Unlike B-2 B cells, B-1 B cells develop in the embryonic yolk sac, self-renew independently of T cell help, and only transiently circulate outside the body cavities (34). Having observed an increase in the overall peritoneal latent reservoir of Cre-positive mice (Fig. 2D), peritoneal cells were pooled from three to five mice/group in each experiment and sorted into B cell (CD19^+^) and other (non-B cell, CD19^−^) populations, with the frequency of MHV68 DNA-positive cells assessed. The frequency of MHV68-positive peritoneal B cells was increased 4.5-fold in Cre-positive as compared to Cre-negative mice (Fig. 5). Consistent with our previous study (34), MHV68 infection was barely detectable in peritoneal non-B cells, and these low levels of infection were not affected by the Cre genotype (Fig. 5). Thus, in contrast to that observed for splenic B cells, B cell-intrinsic STAT1 expression attenuated latent infection of peritoneal B cells.

### Mouse model of B cell-intrinsic type I IFN receptor deficiency

Our studies above revealed a surprising combination of proviral and antiviral functions of B cell-intrinsic STAT1 expression during chronic MHV68 infection. STAT1 was targeted because of its role as a critical antiviral effector of all IFN signaling. However, an important caveat of STAT1-based mouse models is that STAT1 also participates in alternative signaling pathways mediated by select non-IFN cytokines, such as IL-6 (64).

**Fig 6:**
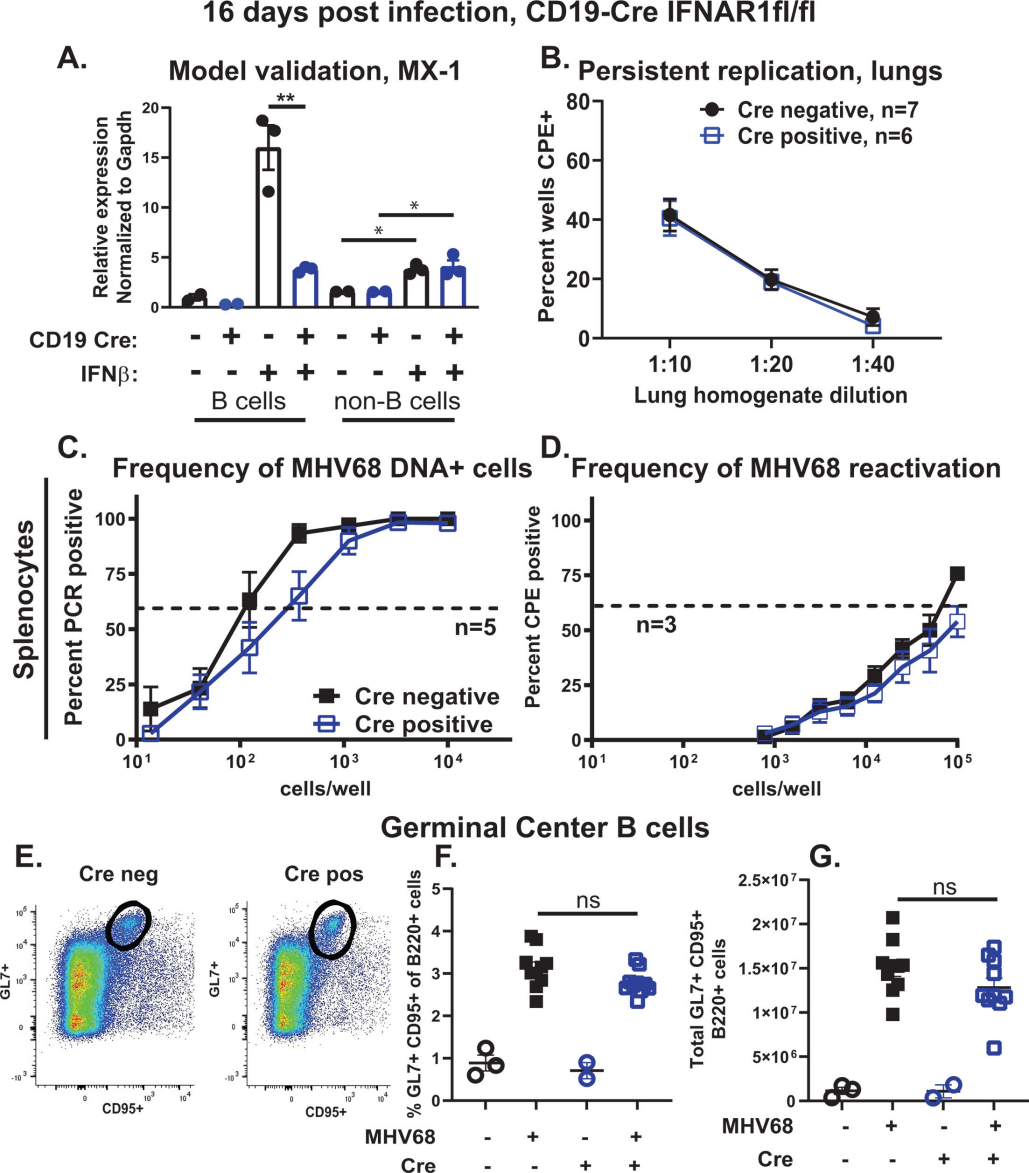
B cell-specific attenuation of type I IFN signaling does not fully phenocopy splenic viral and host phenotypes driven by B cell-specific STAT1 deficiency during chronic MHV68 infection. (A) CD19-Cre IFNAR1^fl/fl^ mouse model validation. Splenocytes isolated from naïve IFNAR1^fl/fl^ mice of the indicated CD19 Cre genotypes were sorted into CD19^+^ B and CD19^−^ non-B cells using magnetic beads. Sorted populations were incubated for 4 hours in the presence or absence of 10 U/mL of recombinant mouse IFNβ. At the end of incubation, RNA was isolated and subjected to quantitative reverse transcription-PCR (qRT-PCR) to measure mRNA levels of MX-1, with subsequent normalization to corresponding GAPDH mRNA levels. Each symbol represents an individual spleen. ^**^P < 0.01; ^*^P < 0.05. (B–G) CD19 Cre-negative and -positive IFNAR1^fl/fl^ littermates were intranasally infected with 10,000 PFU of wild-type MHV68 and analyzed at 16 days post-infection. (B) Persistent MHV68 replication in lung homogenates. Data were pooled from indicated number of animals per group. (C, D) Splenocytes were pooled from three to five animals/group in each experiment and cell suspensions subjected to limiting dilution assays to define the frequency of MHV68 DNA+ cells (**C**) and frequency of *ex vivo* reactivation (**D**). Data in (C, D) were pooled from the indicated number of independent experiments. In the limiting dilution assays, the dotted line is drawn at 63.2%, and the *x*-coordinate of intersection of this line with the sigmoid graph represents an inverse of frequency of positive events. (E–G) Splenic germinal center B cells defined as B220^+^GL7^+^CD95^+^ [representative flow plot of MHV68-infected mice in (E)] were measured by flow cytometry with proportion (**F**) and absolute cell number (**G**) shown. Each symbol in (F, G) represents an individual animal. ns, not significant.

Both type I and type II IFNs are expressed during chronic MHV68 infection (41, 65). Importantly, B cells can express and respond to both type I and II IFNs. To define the extent to which lack of B cell-specific type I IFN signaling was responsible for the viral and host phenotypes observed in the B cell-specific model of STAT1 deficiency, we generated a mouse model where type I IFN signaling was specifically disrupted in B cells. To achieve this, the CD19 Cre recombinase knock-in allele was combined with conditional *Ifnar1* alleles (66) to generate CD19^Cre/wt^*Ifnar1*^fl/fl^ and CD19^wt/wt^ *Ifnar1*^fl/fl^ littermates (referred to as Cre positive and negative). B cell-specific IFNAR1 deficiency did not affect baseline levels of splenic or peritoneal B cell populations (Fig. S1 and S2). To test the extent to which type I IFN signaling was disrupted in B cells, splenocytes from naïve Cre-negative and Cre-positive mice were magnetically sorted into CD19^+^ (B cell) and CD19^−^ (non-B cells) populations with sorted cells cultured in the presence or absence of recombinant mouse IFNβ. Interestingly, splenic B cells sorted from control Cre-negative mice demonstrated a more robust response to type I IFN treatment as compared to the corresponding non-B cell splenocytes (reflected by the increase in the mRNA levels of MX-1, a type I IFN-driven ISG; Fig. 6A). This type I IFN-driven increase in MX-1 mRNA levels was attenuated in splenic B cells isolated from CD19 Cre-positive mice. As expected, IFNβ-induced Mx1 expression was similar in non-B cells isolated from spleens of Cre-negative and Cre-positive mice, validating the B cell-specific model of type I IFN signaling deficiency.

### B cell-specific attenuation of type I IFN signaling does not fully phenocopy splenic viral and host phenotypes driven by B cell-specific STAT1 deficiency during chronic MHV68 infection

Having validated the mouse model of B cell-specific IFNAR1 deficiency, viral and host phenotypes observed in MHV68-infected mice with B cell-specific STAT1 deficiency were examined in the IFNAR1 conditional model. Unlike the decrease in persistent MHV68 lung replication observed in mice lacking STAT1 in B cells (Fig. 2A), infectious MHV68 levels in the lungs were not altered by the B cell-specific IFNAR1 deficiency at 16 days post-infection (Fig. 6B). B cell-intrinsic IFNAR1 deficiency resulted in a 2.2-fold decrease in the frequency of MHV68-positive splenocytes (Fig. 6C, compare to 4.6-fold decrease observed in mice with B cell-intrinsic STAT1 deficiency, Fig. 2B) and did not affect the frequency of *ex vivo* MHV68 reactivation from the spleen (Fig. 6D).

Consistent with the splenic latent reservoir, B cell-specific IFNAR1 deficiency did not impact the proportion or the absolute number of germinal center B cells following MHV68 infection (Fig. 6E through G, gating strategy as in Fig. S3). Similarly, MHV68-driven increase in T follicular helper cells was independent of the CD19 Cre genotype of the IFNAR1 conditional mice (Fig. S4D and E). Finally, the frequencies and numbers of MHV68.ORF73βla-infected germinal center B cells and total splenic B cells were similar in Cre-negative and Cre-positive mice at 16 days post-infection (Fig. S5). In summary, B cell-intrinsic IFNAR1 expression did not fully phenocopy the proviral effects of B cell-intrinsic STAT1 expression on the establishment of MHV68 splenic latency or virus-driven germinal center response.

### B cell-specific type I IFN receptor deficiency does not significantly affect tissue-specific IFN signaling during chronic MHV68 infection

**Fig 7:**
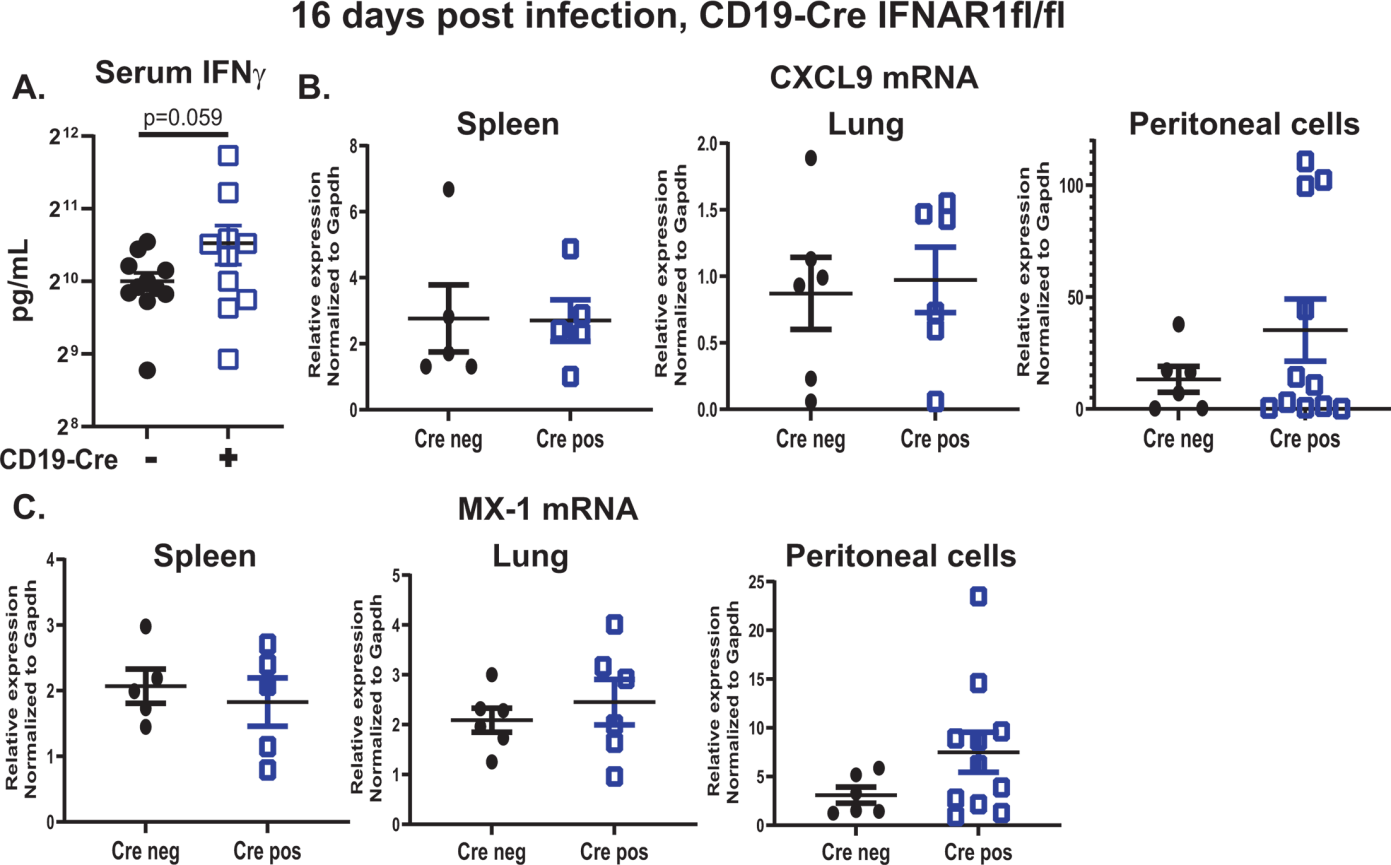
B cell-specific type I IFN receptor deficiency does not attenuate circulating IFNγ levels and does not significantly affect tissue-specific IFN signaling during chronic MHV68 infection. CD19 Cre-negative and -positive IFNAR1^fl/fl^ littermates were infected as in Fig. 6 and analyzed at 16 days post-infection. (A) Serum IFNγ levels measured by ELISA in MHV68-infected mice. (B, C) RNA was isolated at 16 days post-infection from individual lungs, spleens, or peritoneal cavity cells of indicated Cre genotypes and subjected to qRT-PCR to measure relative mRNA levels of CXCL9 (**B**) or MX-1 (**C**) normalized to the corresponding GAPDH mRNA levels. Each symbol represents an individual MHV68-infected animal.

There is a substantial overlap in gene expression driven by type I and II IFNs (67). Furthermore, both type I and type II IFNs are expressed during chronic MHV68 infection (41, 68). Having observed no change in MHV68-driven germinal center response and a 2.2-fold decrease in the splenic latent reservoir of mice with B cell-specific IFNAR1 deficiency, type II IFN levels were assessed as type II IFN could compensate for the attenuated type I IFN signaling in B cells. Interestingly, circulating IFNγ levels appeared to increase in Cre-positive as compared to Cre-negative MHV68-infected mice, although the difference did not reach statistical significance (*P* = 0.059, Fig. 7A).

Systemic IFN levels do not always reflect the IFN concentration in the tissues, which can vary in a tissue-dependent manner. Furthermore, it is the induction of ISGs, and not the mere presence of IFN, that represents the biologically relevant effector function. Thus, ISG levels were measured in tissues examined throughout this study to assess the local IFN effector function. The levels of CXCL9 mRNA, an ISG primarily driven by type II IFN, were similar in the spleens and the lungs of Cre-negative and Cre-positive MHV68-infected mice (Fig. 7B). Interestingly, the mRNA levels of CXCL9 trended increased in peritoneal cells isolated from MHV68-infected Cre-positive as compared to Cre-negative mice; however, the difference did not reach statistical significance (*P* = 0.139). Levels of MX-1 mRNA, a type I IFN-driven ISG, were similar in the spleens and lungs of Cre-negative and -positive MHV68-infected mice (Fig. 7C). However, and similar to that observed for CXCL9 expression in peritoneal cells, the levels of MX-1 mRNA trended increased in the peritoneal cells of MHV68-infected Cre-positive as compared to Cre-negative mice, although the statistical significance was not reached (*P* = 0.0733). Thus, B cell-specific IFNAR1 deficiency did not lead to attenuation and may have led to a selective increase in the tissue-specific IFN signaling during chronic MHV68 infection.

### B cell-specific IFNAR1 expression attenuates MHV68 latency in peritoneal B cells

**Fig 8:**
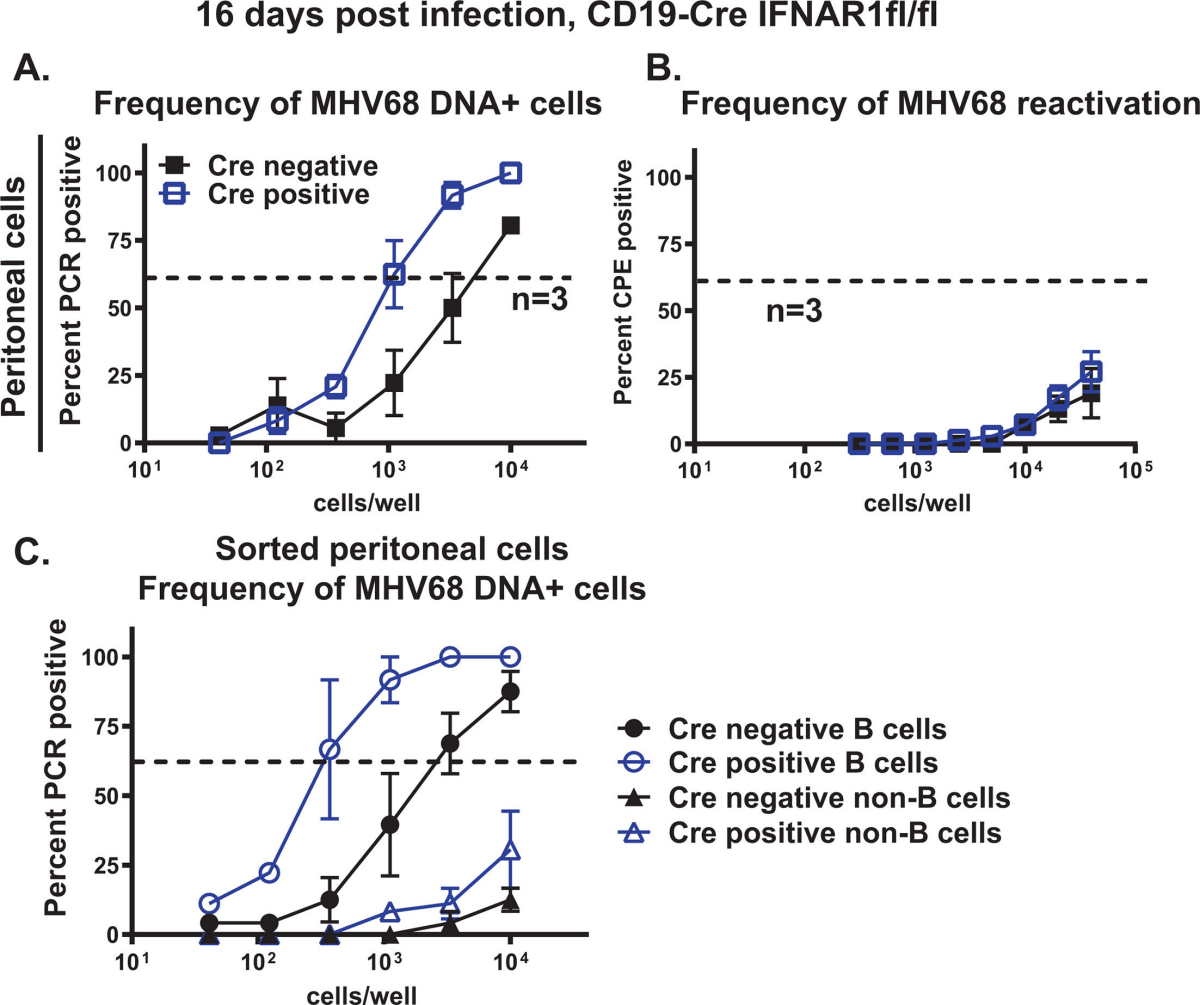
B cell-specific IFNAR1 expression attenuates MHV68 latency in peritoneal B cells. CD19 Cre-negative and -positive IFNAR1^fl/fl^ littermates were infected as in Fig. 6 and analyzed at 16 days post-infection. Peritoneal cells were pooled from five animals/group in each experiment and cell suspensions subjected to limiting dilution assays to define the frequency of MHV68 DNA+ cells (**A**) and frequency of *ex vivo* reactivation (**B**). Data were pooled from the indicated number of independent experiments. (C) Pooled peritoneal cells were sorted into CD19^+^ B cells and CD19^−^ non-B cells using magnetic beads, and sorted populations were subjected to limiting dilution PCR assays. Data were pooled from two to five experiments. The dotted line is drawn at 63.2%, and the *x*-coordinate of intersection of this line with the sigmoid graph represents an inverse of frequency of positive events.

Having assessed the effects of B cell-specific IFNAR1 deficiency on the establishment of MHV68 latency in the spleen, the peritoneal latent reservoir was measured next. Interestingly, and similar to that observed in mice with B cell-specific STAT1 deficiency (Fig. 2 and 5), B cell-specific IFNAR1 deficiency resulted in a 4.4-fold increase in the frequency of MHV68 DNA-positive peritoneal cells at 16 days post-infection (Fig. 8A, compare to the 3.6-fold increase observed in the mouse model of B cell-specific STAT1 deficiency, Fig. 2D). Similarly, low levels of MHV68 reactivation were observed in Cre-negative and Cre-positive groups (Fig. 8B). The increase in MHV68 latent reservoir in peritoneal cells of Cre-positive mice was due to the increased frequency of infection in peritoneal B cells (Fig. 8C). Thus, similar to that observed for the B cell-specific STAT1 expression, B cell-specific IFNAR1 expression was necessary to attenuate the establishment of MHV68 latency in the peritoneal B cells.

## DISCUSSION

IFN signaling is classically defined as a major antiviral system of the host, both at the cellular and systemic levels. Mouse models with global deficiencies have been an invaluable tool for *in vitro* and *in vivo* studies that defined the antiviral role of IFN signaling. However, an important caveat of the global IFN deficiency models is the dramatically altered pathogenesis of virus infection. In the case of herpesvirus infections, including MHV68, high levels of uncontrolled lytic replication and decreased host survival preclude the understanding of the cell type-specific effects of IFN signaling, especially in the context of the latent viral life cycle. In this study, we have used a mouse model of B cell-intrinsic STAT1 deficiency to uncover an intriguing combination of proviral and antiviral roles of STAT1 during chronic gammaherpesvirus infection of an intact natural host. Interestingly, B cell-specific IFNAR1 deficiency fully recapitulated the antiviral but not the proviral phenotypes observed in the STAT1 conditional model during chronic gammaherpesvirus infection.

### Unexpected proviral role of B cell-intrinsic STAT1 expression in chronic MHV68 infection

Our study is the first to demonstrate the proviral role of B cell-specific STAT1 expression during the establishment of chronic MHV68 infection in the spleen, including support of latent infection of germinal center B cells that host the majority of the MHV68 splenic latent reservoir at 16 days post-infection. Interestingly, B cell-specific STAT1 expression selectively promoted the generation of self-reactive but not MHV68-specific class-switched antibodies driven by MHV68 infection. Similar phenotypes were previously observed in a model of spontaneous B cell-driven autoimmunity, where B cell-specific IFNγ receptor expression was required for the generation of autoimmune B cell responses and disease but was dispensable for physiological B cell responses following immunization (69). In contrast, B cell-specific IFNAR1 expression accelerated the kinetics but was not required for the generation of autoimmune disease on the same autoimmune background (69).

Interestingly, in our current study, the splenic MHV68 and host phenotypes observed during chronic infection of mice with B cell-specific STAT1 deficiency were not fully phenocopied in mice with B cell-specific deficiency of type I IFN receptor. Given that STAT1 participates in signaling downstream of non-IFN cytokines, it is possible that the viral and host phenotypes driven by B cell-specific STAT1 expression are reflective of the role of STAT1 in other cytokine signaling pathways, such as IL-6, a cytokine that promotes B cell differentiation (64). However, a more likely hypothesis is that B cell-specific IFNAR1 deficiency was compensated by the B cell-intrinsic STAT1 activation by IFNγ in the CD19 Cre-positive *Ifnar1^fl/fl^* MHV68-infected mice. Future studies will employ a mouse model of combined B cell-specific IFNAR1 and IFNγR deficiency to define the extent to which B cell-intrinsic IFN signaling is proviral during chronic MHV68 infection of splenic B cells.

In addition to its classical role in IFN signaling, STAT1 promotes the differentiation of select immune cell types. T cell-intrinsic STAT1 expression promotes Th1 and early TFH differentiation of CD4 T cells (70, 71), the latter also observed during chronic MHV68 infection (50). In B cells, STAT1 is important for the differentiation of marginal zone B cells into IgM-producing plasma cells (72). However, IgM titers were not affected by B cell-specific STAT1 deficiency in chronically infected mice (Fig. 3F). Instead, the decrease in the MHV68 latent reservoir observed in mice with B cell-specific STAT1 deficiency was due to attenuated infection of germinal center B cells that are independent of marginal zone B cells in their differentiation trajectory. We observed that B cell-specific STAT1 expression selectively supported the generation of dsDNA-specific but not MHV68-specific class-switched antibodies during chronic infection (Fig. 3G and H). STAT1 and IFNγ signaling are implicated in the differentiation of T-bet-expressing self-reactive memory B cell population, also known as double-negative, atypical memory, or age-associated B cells (ABCs), in mouse autoimmune models (69, 73, 74). Importantly, ABCs are highly pleiotropic, with the function modified by the experimental system. In contrast to the pathogenic roles in self-reactive antibody production in autoimmune models, ABCs are primarily responsible for antiviral-antibody production during influenza infection (75). MHV68 infection drives the increase in the ABC population; however, the role of STAT1 and IFNγ signaling in MHV68-driven ABC expansion has not been tested. While MHV68-driven ABCs are capable of expressing anti-MHV68 antibodies, the overall anti-MHV68 IgG titers are not affected, and MHV68 reactivation from splenocytes is increased in mice with B cell-specific T-bet deficiency (76–78), suggesting that T-bet-expressing ABCs are antiviral in the context of MHV68 infection. Thus, the involvement, if any, of ABCs in the proviral STAT1 phenotypes observed in this study remains unclear and requires additional investigation.

### Opposing roles of STAT1 in B-1 vs B-2 B cells during MHV68 chronic infection

Our current study demonstrates that while B cell-intrinsic STAT1 expression supports the latent reservoir in B-2 B cells of the spleen, it attenuates the latent reservoir in peritoneal B cells. Peritoneal B cells are represented by two subpopulations of B-1 B cells (B-1a and B-1b) and a B-2 B cell population. B cells immunophenotyping as B-2 B cells in the peritoneal cavity are thought to acquire many of the characteristics of B-1 B cells, distinguishing those from splenic B-2 B cells (79).

We showed that intranasal inoculation of MHV68 results in the peritoneal B-1b B cells being the major host of the viral latent reservoir in the peritoneal cavity, with considerably lower frequency of latent infection found in peritoneal B-2 B cells and no latent virus found in the B-1a B cell population (34, 35). In contrast, very little if any latent MHV68 infection was found in other peritoneal non-B cell types. Unlike MHV68 reactivation in B-2 B cells in the spleen that is unequivocally tied to plasma cell differentiation, mechanisms that regulate MHV68 reactivation from B-1 B cells remain poorly understood, with B-1 B cells supporting very low levels of MHV68 reactivation, at least on the C57BL/6J genetic background (34).

In this study, we observed an increased frequency of latent infection, but not *ex vivo* reactivation, in peritoneal cells of mice with B cell-specific deficiency of either STAT1 or IFNAR1, implicating B cell-intrinsic type I IFN signaling in the control of the peritoneal latent reservoir. MHV68 has evolved a STAT1 binding element within the gene encoding viral lytic switch transcription factor (orf50), with type I and II IFN signaling and STAT1 activation repressing the activity of the orf50 promoter (80, 81). Viral manipulation of STAT1 to promote herpesvirus latency may be a common goal, as shown by the study from the Goodrum group demonstrating that HCMV-encoded UL138 promotes STAT1 activation to skew the balance between lytic and latent infection toward the latter (82). Thus, it is possible that loss of STAT1 or IFNAR1 in peritoneal B cells leads to increased MHV68 lytic gene expression. However, it is unusual that the hypothesized increase in the expression of viral lytic proteins has not led to increased killing of infected peritoneal B cells by cytotoxic T cells.

Curiously, unlike similar induction of type I and type II IFN-dependent ISGs observed in lungs and spleens of control mice and mice with B cell-specific IFNAR1 deficiency, the ISG expression trended toward increased in the peritoneal cells of mice lacking IFNAR1 expression specifically in B cells (Fig. 7B and C). This could be due to higher type II IFN expression in the peritoneal cavity or qualitative and/or quantitative differences in IFN responses between splenic B-2 and peritoneal B-1 B cell populations. While type I IFN signaling in B-1a B cells mobilizes these cells to mediastinal lymph nodes during influenza infection (83) and facilitates the generation of antibodies against T cell-independent type 2 antigens by B1b-B cells (84), type I and II IFN responses have not been directly compared in B-1 vs B-2 B cells. It is intriguing to speculate that differential type I and II IFN signaling in these two B cell subsets is responsible for the antiviral roles of B cell-intrinsic STAT1 and type I IFN signaling we had observed during chronic MHV68 infection in the peritoneal cavity. Defining the differential outcomes of IFN signaling in B-1 vs B-2 B cells in the context of MHV68 infection is challenging given the current absence of an experimental system allowing robust infection of primary murine B cells by MHV68 *in vitro* and awaits future technical improvements.

## MATERIALS AND METHODS

### Animal studies

All animal studies were approved by the Institutional Animal Care and Use Committee of the Medical College of Wisconsin (MCW) (AUA0971). *Stat1^fl/fl^* mice (51) were a kind gift of Dr. Bonnie Dittel. The B cell-specific mouse model of STAT1 deficiency was generated by crossing *Cd19* Cre recombinase knock-in allele (*Cd19^Cre/wt^*) (52) with *Stat1^fl/fl^* alleles to generate *Cd19^Cre/wt^ Stat1^fl/fl^* and *Cd19^wt/wt^ Stat1^fl/fl^* littermates. *Cd19^Cre/wt^ Ifnar1^fl/fl^* and *Cd19^wt/wt^ Ifnar1^fl/fl^* littermates were generated in a similar fashion using the *Ifnar1^fl/fl^* model (66). *Ifnar1^fl/fl^* and *Cd19^Cre/wt^* mouse strains were obtained from Jackson Laboratories (Bar Harbor, ME). Mice were bred and housed in a specific pathogen-free facility at MCW. At 6–7 weeks of age, Cre-positive and -negative littermates were intranasally infected under light anesthesia with 10,000 PFU of MHV68 diluted in sterile, serum-free Dulbecco’s modified Eagle’s medium immediately prior to infection. MHV68 viral stock was prepared and titered using the 3T12 fibroblast cell line.

### Limiting dilution assays

The frequency of MHV68 DNA-positive cells was determined as previously described (85). Specifically, splenocytes and peritoneal cells were pooled from all mice in each experimental group (three to five mice/group), and six threefold serial dilutions were subjected to nested PCR reactions (12 replicates/dilution) using primers against the viral genome. To determine the frequency of *ex vivo* MHV68 reactivation, splenocytes and peritoneal cells were pooled from all mice within each experimental group (three to five mice/group), and eight twofold serial dilutions of cell suspensions from each group were plated onto a monolayer of MEFs at 24 replicates per dilution. To control for preformed virus, four twofold serial dilutions of mechanically disrupted splenic and peritoneal cells were plated on MEFs. Viral reactivation as indicated by cytopathic clearing of MEFs was assessed on day 21 of culture. Lungs were homogenized (MagNAlyzer, Roche) using 1-mm sterile zirconia beads and plated at 1:10, 1:20, and 1:40 dilutions on MEFs (12 replicates/dilution). Cytopathic clearing of MEFs was scored at 14 days of culture.

### Cell sorting

Peritoneal cells were pooled from all mice within each experimental group (three to five mice/group) at 16 days post-MHV68 infection. B cells were isolated using magnetic beads provided within the EasySep Mouse CD19 Positive Selection Kit (Stemcell Technologies, Vancouver, CA) according to the manufacturer’s instructions. In brief, 1 × 10^8^ peritoneal cells were suspended in 1 mL of sorting media (phosphate-buffered saline, 2% Fetal Bovine Serum (FBS), and 1 mM EDTA). Fifty microliters of selection cocktail was added to the cell suspension followed by a 5-min incubation at room temperature. After incubation, 75 µL of RapidSpheres was added to the cell suspension and incubated at room temperature for 3 min. 1.5 mL of sorting media was then added before placement into EasySep magnet for 3 min. The cells collected in the liquid fraction are considered flow through (non-B cells). The cells mobilized to the tube wall by the magnet (B cells) were resuspended in 1 mL of fresh sorting media prior to analysis. The same experimental approach was used to generate data in Fig. 1A and 6A, where splenocytes isolated from naïve mice of indicated genotypes were separated into CD19^+^ B cell populations (B cells) and remaining splenocytes (non-B cells). For studies presented in Fig. 1A , cells were immediately lysed for western analyses, as below. For studies presented in Fig. 6A, the two cell populations were cultured for 4 hours in the presence or absence of recombinant mouse IFNβ (10 U/mL), with subsequent RNA isolation and quantitative reverse transcription-PCR (qRT-PCR) analyses, as below.

### qRT-PCR analysis

Total RNA was isolated from organs or cell suspensions generated from organs of infected mice, treated with DNase, subjected to reverse transcription, and analyzed by qRT-PCR using the CFX connect system (Bio-Rad, Hercules, CA) as previously described (49). cDNA was amplified using CXCL9 forward (5′-GCTCTAGAGAGGAGGTCTGATG-3′) and reverse (5′-AGGCTTTGGCTAGTCGTTATG-3′) primers, MX-1 forward (5′- AGCTAGACAGAGCAAACCAAGCCA-3′) and reverse (5′- TCCCTGAAGCAGACACAGCTGAAA-3′), and GAPDH forward (5′- TGTGATGCGTGTGAACCACGAGAA-3′) and reverse (5′- GAGCCCTTCCACAATGCCAAAGTT-3′) primers previously validated for specificity and used within the corresponding linear range (49). Minus RT reactions were used as controls for genomic DNA contamination.

### Flow cytometry

Single-cell suspensions of splenocytes or peritoneal cells were prepared in fluorescence-activated cell sorting buffer (phosphate-buffered saline, 2% fetal bovine serum). 2 × 10^6^ cells were prestained with Fc block and then incubated with optimized antibody concentrations. Data were acquired using Celesta flow cytometer (BD Biosciences, Franklin Lakes, NJ), and data were analyzed using FlowJo software (BD Life Sciences, Franklin Lakes, NJ). The following antibodies used in this study were purchased from BioLegend (San Diego, CA): B220-PECy7 (cat. 103222), CD19-PB (cat. 115523), GL7-APC (cat. 144617), GL7-FITC (cat. 144604), CD95-APC (cat. 152604), CD3-PB (cat. 100214), CD4-FITC (cat. 100406), PD-1-BV605 (cat. 135220), CXCR5-PE/Dazzle 594 (cat. 145522), IgG-APC, IgM-FITC (cat. 406505), IgD-PB (cat. 405711), and IRF-4-PE (cat. 646404); or BD Biosciences (Franklin Lakes, NJ): CD95-PE-CF594 (cat. 562395). Cells infected with the MHV68.ORF73BLa reporter virus were detected using the LiveBLAzer FRET-B/G Loading Kit with CCF2-AM (cat. K1032) from Thermo Fisher Scientific (Waltham, MA).

### Enzyme-linked immunosorbent assays

ELISAs to measure serum IgM, IgG, MHV68-specific IgG, and anti-double-stranded DNA IgG were performed as previously described (86).

### Western blot analyses

After magnetic cell sorting (above), 6 × 10^6^ splenic B cells were lysed in radioimmunoprecipitation assay buffer containing protease inhibitors (1 µg/mL pepstatin/leupeptin/aprotinin and 20 µg/mL phenylmethylsulfonyl fluoride), and total protein concentrations were determined by the Lowry method (80). Cellular lysates (10–20 μg protein) were separated by 8% or 10% SDS-PAGE and wet-transferred to nitrocellulose membranes. The membranes were blocked with 5% non-fat milk (wt/vol) in 1× Tris-buffered saline with 0.05% Tween-20 (TBST) at room temperature for 1 hour and then probed with anti-total STAT1 (1:10,000, Santa Cruz Biotechnology, Dallas, TX) and anti-beta actin (1:10,000, Cell Signaling Technology, Danvers, MA, cat. 4970) antibodies in 1% milk in TBST overnight at 4°C. Membranes were washed and incubated with horseradish peroxidase-conjugated anti-rabbit-IgG secondary antibody (1:15,000) in 1% milk in TBST for 1 hour at room temperature. The membranes were washed, and SuperSignal West Pico PLUS (Thermo Fisher Scientific, Waltham, MA) chemiluminescent substrate was allowed to react with the membranes for 5 min before blots were imaged using a ChemiDoc MP.

### Statistical analyses

Statistical analyses were performed using the Student *t*-test (Prism, GraphPad Software, Inc.).

## SUPPLEMENTAL MATERIAL

Fig. S1 mbio.01598-24-s0001.eps Splenic B cell immunophenotyping.

Fig. S2 mbio.01598-24-s0002.eps Peritoneal cavity B cell immunophenotyping.

Fig. S3 mbio.01598-24-s0003.eps Flow cytometry gating strategy for germinal center B cells.

Fig. S4 mbio.01598-24-s0004.eps Measurement of MHV68-induced T follicular helper cells.

Fig. S5 mbio.01598-24-s0005.eps B cell-specific IFNAR1 deficiency does not affect the establishment of MHV68 latency in germinal center B cells.

ASM does not own the copyrights to Supplemental Material that may be linked to, or accessed through, an article. The authors have granted ASM a non-exclusive, world-wide license to publish the Supplemental Material files. Please contact the corresponding author directly for reuse.
